# Mechanism of PGC-1α-mediated mitochondrial biogenesis in cerebral ischemia–reperfusion injury

**DOI:** 10.3389/fnmol.2023.1224964

**Published:** 2023-07-10

**Authors:** Ying Yuan, Yuan Tian, Hui Jiang, Luo-yang Cai, Jie Song, Rui Peng, Xiao-ming Zhang

**Affiliations:** ^1^School of Acupuncture-Moxibustion and Orthopedics, Hubei University of Chinese Medicine, Wuhan, China; ^2^Sub-Health Institute Hubei University of Chinese Medicine, Wuhan, China; ^3^Hubei Provincial Collaborative Innovation Center for Preventive Treatment of Disease by Acupuncture, Wuhan, China

**Keywords:** cerebral ischemia–reperfusion injury, mitochondrial biogenesis, mitochondria, PGC-1α, apoptosis

## Abstract

Cerebral ischemia–reperfusion injury (CIRI) is a series of cascade reactions that occur after blood flow recanalization in the ischemic zone in patients with cerebral infarction, causing an imbalance in intracellular homeostasis through multiple pathologies such as increased oxygen free radicals, inflammatory response, calcium overload, and impaired energy metabolism, leading to mitochondrial dysfunction and ultimately apoptosis. Rescue of reversibly damaged neurons in the ischemic hemispheric zone is the key to saving brain infarction and reducing neurological deficits. Complex and active neurological functions are highly dependent on an adequate energy supply from mitochondria. Mitochondrial biogenesis (MB), a process that generates new functional mitochondria and restores normal mitochondrial function by replacing damaged mitochondria, is a major mechanism for maintaining intra-mitochondrial homeostasis and is involved in mitochondrial quality control to ameliorate mitochondrial dysfunction and thus protects against CIRI. The main regulator of MB is peroxisome proliferator-activated receptor gamma coactivator-1α (PGC-1α), which improves mitochondrial function to protect against CIRI by activating its downstream nuclear respiratory factor 1 (NRF1) and mitochondrial transcription factor A (TFAM) to promote mitochondrial genome replication and transcription. This paper provides a theoretical reference for the treatment of neurological impairment caused by CIRI by discussing the mechanisms of mitochondrial biogenesis during cerebral ischemia–reperfusion injury.

## 1. Introduction

Cerebral ischemia is a major health problem worldwide, with a high morbidity rate and a very high rate of disability and mortality (Lapchak and Zhang, 2017; Yang et al., 2018). An ischemic stroke consists of two related pathological injury processes: primary ischemia-induced brain injury and secondary ischemia/reperfusion (I/R) injury. An effective approach after cerebrovascular embolism in clinical practice is timely thrombolysis and restoration of blood perfusion in the ischemic area. However, blood flow recanalization after cerebral ischemia can cause unavoidable damage to brain tissue through a series of pathological reactions, namely cerebral ischemia–reperfusion injury (CIRI). After CIRI, the necrotic core is surrounded by a peri-infarct area called the “ischemic penumbra,” which is functionally impaired but still viable, and the damaged neurons in the penumbra may be salvageable with post-stroke treatment (Geng et al., 2022). Recent studies have shown that neurons in the ischemic penumbra may undergo apoptosis hours or days after ischemia, and that intervention in the penumbra to stop or inhibit the apoptotic process is an achievable therapeutic goal aimed at limiting the amount of infarction after clinical stroke (Uzdensky, 2019). Mitochondria, as the energy factories of cells, can undergo a series of damaging changes during ischemia and hypoxia, which can lead to pathological abnormalities such as insufficient energy supply and induction of neuronal apoptosis, and are the key subcellular basis of neurological dysfunction in patients with cerebral infarction. Intervention during this period to promote mitochondrial biosynthesis to inhibit apoptosis is an achievable therapeutic goal.

Mitochondrial homeostasis is composed of mitochondrial fusion and fission, degradation of dysfunctional mitochondria through mitochondrial autophagy or the ubiquitin-proteasome system, and generation of new mitochondria from the existing mitochondrial pool through mitochondrial biogenesis (MB) (Liu et al., 2018). Mitochondrial biosynthesis contributes to the replacement and repair of damaged mitochondria in neurons, and enhanced mitochondrial biosynthesis may be a novel pathway for neurological recovery after CIRI. Therefore, elucidating the correlation between mitochondrial biosynthesis and CIRI and using mitochondrial biosynthesis-related proteins as a new target for studying CIRI are important for further exploring the mechanistic studies of CIRI.

## 2. Mechanism of action of cerebral ischemia–reperfusion injury

**Figure 1:**
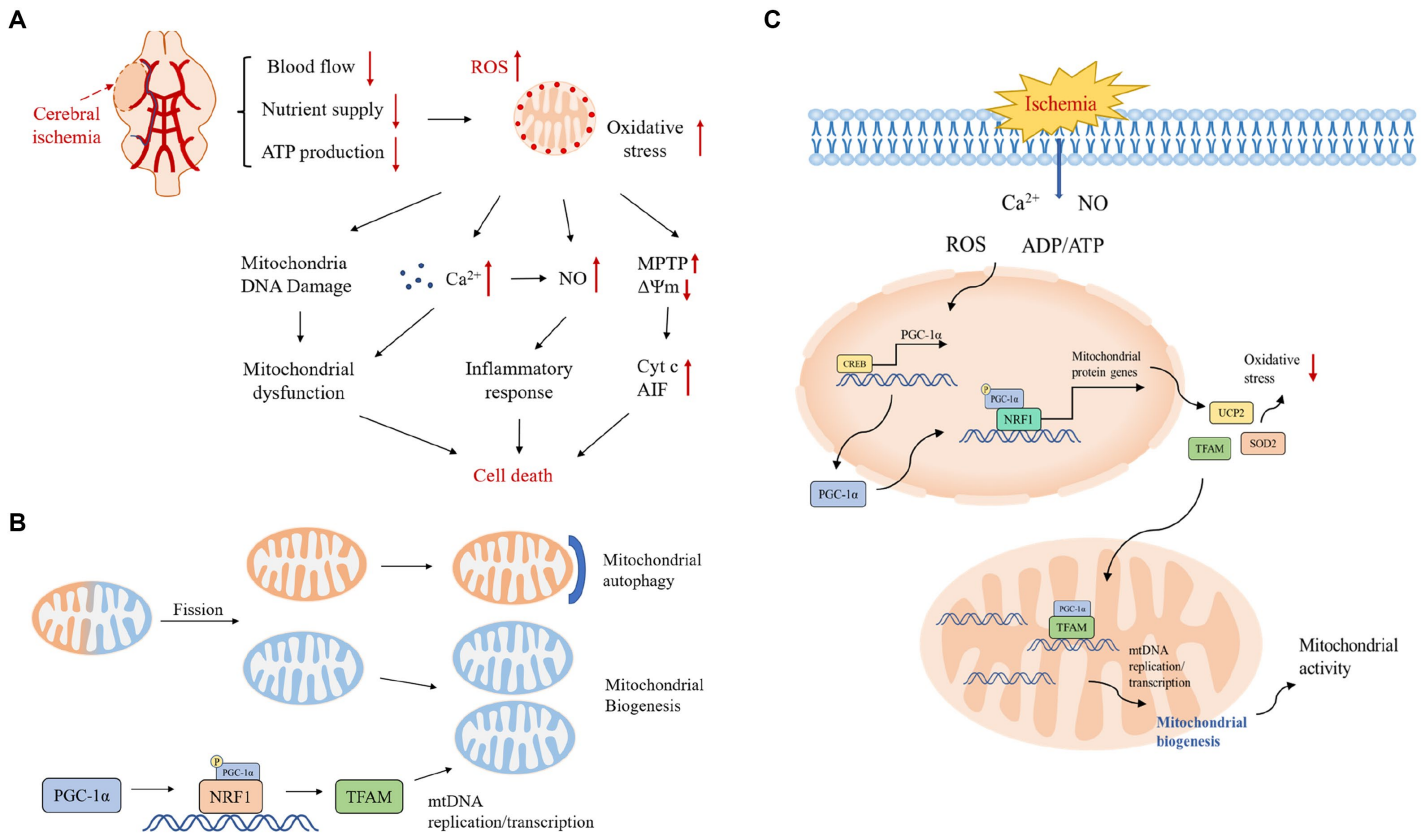
**(A)** Pathological mechanisms in the cerebral ischemic cascade response. Excessive production of reactive oxygen species after cerebral ischemia activates various downstream pathological processes, excessive Ca^2+^ influx, mitochondrial DNA damage leading to mitochondrial dysfunction, activation of inflammatory factors inducing an inflammatory response, and under stressful conditions, transient opening of MPTP in the mitochondrial inner membrane leading to collapse of the mitochondrial transmembrane potential and triggering the release of Cyt c and other pro-apoptotic molecules, which together initiate the apoptotic cascade reaction. ΔΨm, mitochondrial transmembrane potential; Cyt c, cytochrome c; NO, nitric oxide. **(B)** Mitochondrial quality control mechanisms. Mitochondrial quality control includes the dynamic balance of mitochondrial autophagy, biogenesis, fusion and fission. Mitochondrial autophagy and biogenesis are regulated in a coordinated manner to replace damaged mitochondria during periods of high mitochondrial turnover. When mitochondrial function is dysfunctional under hypoxia, unhealthy components of mitochondria will fission from the healthy mitochondrial network and then degrade to fragments through autophagy; the remaining healthy mitochondrial network can grow and divide through biogenesis. **(C)** Protective mechanisms of PGC-1α during ischemia-induced stress and a central role in mitochondrial biogenesis. The stress-inducible molecules ROS, Ca^2+^, ADP/ATP and NO promote the expression of PGC-1α, which upregulates the expression of antioxidant proteins and increases mitochondrial biogenesis to protect neurons from oxidative stress and promote neuronal survival.

Cerebral ischemia–reperfusion injury is a complex process, and after ischemia and hypoxia, blood flow recanalization in brain tissue triggers a series of pathological responses including increased oxygen free radicals, inflammatory response, calcium overload, impaired energy metabolism, and apoptosis, among other processes (Figure 1A; Manzanero et al., 2013; al-Mufti et al., 2018). Mitochondria play a key role in brain injury, and mitochondrial dysfunction is a key factor in the pathogenesis of CIRI, which can lead to neuronal dysfunction and cell death in the brain.

### 2.1. Oxidative stress

Oxidative stress is one of the main mechanisms of CIRI. Under normal conditions, moderate amounts of reactive oxygen species (ROS) play an important role in several physiological processes and they can be eliminated by endogenous antioxidant systems (Liang et al., 2017). In patients with cerebral ischemia, a significant decrease in local cerebral blood flow leads to abnormal function of neurons in the ischemic area and ultimately to brain tissue damage. After reperfusion, the restoration of blood flow and oxygen, although essential to maintain neuronal viability, allows mitochondria to release large amounts of oxygen radicals through their electron transport chain, generating large amounts of ROS in neurons (Chen et al., 2011; Andrabi et al., 2020); post-IR hypoxia-inducible factors likewise activate pro-oxidant enzymes and generate more ROS. Excess reactive oxygen species generate toxic oxygen derivatives such as superoxide anions and hydrogen peroxide, which can eventually lead to neuronal damage through the apoptotic pathway (Lennon et al., 1991). After CIRI, the balance between ROS production and clearance is compromised, which may lead to oxidative stress-induced membrane lipid peroxidation, DNA damage, and mitochondrial dysfunction, ultimately leading to neuronal apoptosis and necrosis, resulting in irreversible damage to brain tissue (Ham and Raju, 2017; Jia et al., 2021). Peroxisome proliferator-activated receptor gamma coactivator-1α (PGC-1α) plays a central role in protective mechanisms and mitochondrial biogenesis during ischemia-induced stress, and stress-inducing molecules such as ROS promote the expression of PGC-1α, which protects neurons from oxidative stress by upregulating the expression of antioxidant proteins and enhancing mitochondrial biogenesis to reduce differences in oxygen release before and after cerebral ischemia (Abu Shelbayeh et al., 2023).

### 2.2. Inflammatory response

Inflammatory cells are rapidly activated after CIRI, and glial cells are considered to be one of the key factors triggering the inflammatory response. Inflammatory effector cells infiltrate and then release large amounts of inflammatory mediators to cause local brain tissue edema eventually leading to tissue injury (Shi et al., 2019; Xu et al., 2020). After the stroke, damaged tissues produce large amounts of ROS and pro-inflammatory mediators such as cytokines and chemokines, which induce leukocyte aggregation and infiltration at the site of cerebral ischemia; leukocyte aggregation can then release inflammatory cytokines and mediators, which induce more leukocyte aggregation and infiltration and aggravate the inflammatory response in CIRI (Wanrooy et al., 2021). Post-stroke polarized microglia activate inflammatory response pathways and increase the expression of inflammatory factors such as interleukin-1 (IL-1) and interleukin-6 (IL-6), as well as astrocytes and leukocytes, which can increase the synthesis of inflammatory cytokines and release pro-inflammatory factors in ischemic areas, followed by activating the transcription of multiple inflammation-related genes and increasing the expression of inflammatory factors, exacerbating the brain inflammatory response, leading to neuronal death (Pantoni et al., 1998; Levard et al., 2021). Altered PGC-1α levels after stroke disrupt the balance of macrophage types during inflammation, and overexpression of PGC-1α reduces the expression of pro-inflammatory cytokines such as microglia, suggesting that PGC-1α-induced mitochondrial biosynthesis alleviates mitochondrial dysfunction in CIRI by suppressing the inflammatory response and ameliorates neuronal damage to protect neurological function (Cherry and Piantadosi, 2015).

### 2.3. Calcium overload

Under normal conditions, the extracellular calcium ion concentration is much higher than the intracellular concentration, and after CIRI occurs, various injuries such as oxidative stress can alter cell membrane ion channels and membrane permeability, resulting in massive calcium inward flow and excessive intracellular calcium content, causing multiple abnormalities such as neuronal structural damage and cerebrovascular dysfunction, and ultimately activating apoptotic pathways in neurons in the ischemic area (Ma et al., 2017; Zhang et al., 2019; Ludhiadch et al., 2022). In the case of calcium overload, calmodulin (CaM) binds to Ca^2+^ in a complex increasing the release of vasoconstrictor factors, causing vasospastic constriction affecting blood flow and aggravating the ischemic–hypoxic injury after CIRI. Calcium overload also increases the synthesis of free fatty acids by activating phospholipases A and C, which disrupts the neuronal skeleton and leads to cellular edema (Peng-Fei et al., 2021). In addition, intracellular Ca^2+^ overload increases the passive uptake of calcium by mitochondria, resulting in mitochondrial dysfunction due to the deposition of calcium phosphate and reduced energy production in mitochondria (Mao et al., 2022; Tuo et al., 2022). It was also found that functional defects in the associated complexes on the inner mitochondrial membrane trigger mitochondrial dysfunction, leading to dysregulation of intracellular Ca^2+^ homeostasis and increased cytoplasmic levels of Ca^2+^, suggesting an interaction mechanism between Ca^2+^ homeostasis and mitochondrial dysfunction (Wang and Wei, 2017).

### 2.4. Apoptosis

Mitochondrial dysfunction also plays an important role in the mechanism of neuronal apoptosis. After CIRI, a large amount of ROS produced by mitochondria can trigger a variety of pathological signaling pathways, such as mitochondria-dependent apoptosis, DNA damage response, and inflammatory response, in addition to directly damaging lipids, proteins and nucleic acids in cells (Chen et al., 2022). Among them, apoptosis can be initiated by damage to mitochondria, followed by induction of cell death through the release of pro-apoptotic proteins such as cytochrome c or apoptosis-inducing factor (Niizuma et al., 2010). The cell death process is critically dependent on the intracellular ATP concentration, as ATP production depends on the structural and functional integrity of mitochondria.The transient opening of the mitochondrial permeability transition pore (MPTP) in the inner mitochondrial membrane is induced by various cellular stress conditions, which leads to the collapse of the mitochondrial transmembrane potential initiates an apoptotic cascade response, which triggers the release of cytochrome c and other pro-apoptotic molecules and ultimately initiates the apoptotic process leading to neuronal death (Anzell et al., 2018; Cao et al., 2021; Zhang et al., 2022).

## 3. Mitochondrial biogenesis

Mitochondria are DNA-containing subcellular organelles that generate energy to supply the cell through oxidative phosphorylation (Roger et al., 2017). Mitochondria can respond to the changing cellular environment through quality control to ensure mitochondrial homeostasis, which is essential for mitochondria to maintain their normal function, structure and motility (Fu et al., 2019). Mitochondria can remove damaged mitochondria and replenish healthy ones through mitochondrial biogenesis, mitochondrial autophagy, mitochondrial fusion and fission (Figure 1B; Seo et al., 2018). Mitochondrial dynamics including fusion and fission play an important role in maintaining mitochondrial function and morphology. When mitochondrial function is dysfunctional under hypoxia, unhealthy components of mitochondria will fission from the healthy mitochondrial network and then degrade into fragments through autophagy; certain damaged mitochondria can fuse with other healthy mitochondria to maintain a healthy mitochondrial network, suggesting a crucial role of mitochondrial dynamics in ischemic neuronal injury and recovery. The mitochondrial quality control system is essential for maintaining a functional mitochondrial population in neurons and relies on the ubiquitin proteasome system (UPS) and mitochondrial autophagy to remove senescent and dysfunctional mitochondria, and mitochondrial autophagy during IR can play its protective role by removing damaged mitochondria and inhibiting downstream apoptosis (Pickles et al., 2018; Evans and Holzbaur, 2020). Mitochondrial biosynthesis is the growth of the existing pool of healthy mitochondria, and after the degradation of damaged mitochondria, the existing mitochondria need to continue to grow to keep up with the energy demands of the cell. Therefore, a strict mitochondrial quality control mechanism is a critical process to protect mitochondrial quality and function as well as determine cell fate and is essential to orchestrate a healthy mitochondrial network.

Mitochondrial biosynthesis is a complex process involving several different processes: (1) synthesis of the inner mitochondrial membrane (IMM) and outer mitochondrial membrane (OMM) (2) synthesis of mitochondrial-encoded proteins (3) synthesis and import of nuclear-encoded mitochondrial proteins; and (4) replication of mtDNA (Uittenbogaard and Chiaramello, 2014). Mitochondrial biogenesis requires coordinated regulation of the nuclear and mitochondrial genomes, as most mitochondrial proteins are encoded by nuclear genes (Gureev et al., 2019; Sharma and Sampath, 2019). PGC-1α is often considered a key regulator of mitochondrial biogenesis, which is abundantly expressed in metabolically active organs such as the brain, heart, and kidney and can coordinate transcriptional mechanisms to increase mitochondrial mass and thus adapt tissues to increased energy demands (Li et al., 2017). Nuclear respiratory factor 1 (NRF1) is a nuclear transcription factor that acts on nuclear motifs encoding component subunits of the oxidative phosphorylation system (OXPHOS), and NRF1 also regulates the expression of a series of genes involved in mtDNA transcription and replication-related genes (Virbasius C. A. et al., 1993; Virbasius J. V. et al., 1993). Active PGC-1α translocates into the nucleus and activates NRF1, which subsequently transcribes nuclear-encoded respiratory chain components and mitochondrial transcription factor A (TFAM), thereby promoting mitochondrial protein synthesis, mtDNA replication and transcription, and *de novo* mitochondrial biosynthesis (Yin et al., 2008; de Oliveira Bristot et al., 2019). mtDNA is transcribed by mitochondrial RNA polymerase (POLRMT), and the key enhancer protein is TFAM, which ensures the proper mtRNA spreading and bending required for POLRMT to bind to the mtDNA promoter (Kühl et al., 2016; Shokolenko and Alexeyev, 2017).

PGC-1α expression also mediates mitochondrial uncoupling through the induction of proteins such as uncoupling protein-2 (UCP2), an internal mitochondrial membrane protein that dissipates mitochondrial membrane potential to uncouple electron transport from ATP synthesis, thereby reducing ROS production (Hermes et al., 2016). UCP2 has also been shown to reduce pro-inflammatory cytokine production, mitochondrial calcium overload, and the potential of apoptotic events (de Oliveira Bristot et al., 2019). It has been shown that reduced UCP2 expression is associated with mitochondrial dysfunction, ROS accumulation and increased apoptosis; therefore, UCP2 may be involved in the pathogenesis of neurodegenerative diseases, including stroke (Cenini et al., 2019). Studies have shown that activation of the PGC-1α axis also induces the expression of paraoxonase-2 (PON2), another protein with potent antioxidant and neuroprotective properties (Gupta and Costa, 2021). PON2 is highly expressed in the brain and is mainly localized in the mitochondrial and endoplasmic reticulum membranes, and there is evidence that PON2 is essential for normal mitochondrial function and plays an important role in reducing oxidative stress (Devarajan et al., 2018).

In summary, NRF1, UCP2, and PON2 play key roles in PGC-1α-induced mitochondrial biosynthesis, and they improve oxidative stress, reduce apoptosis, and protect normal mitochondrial function by reducing ROS release, updating our current knowledge and understanding of the mechanisms of mitochondrial biosynthesis in PGC-1α-induced cerebral ischemic diseases, so mitochondrial biosynthesis may become a potential new target for CIRI therapy.

## 4. Mitochondrial biogenesis and cerebral ischemia–reperfusion injury

Ischemia–reperfusion (IR) can trigger apoptotic pathways by increasing the production of reactive oxygen species, disrupting calcium homeostasis and inducing an inflammatory response, and mitochondria are affected by post-IR cascade events resulting in dysfunction; mitochondrial dysfunction in turn exacerbates neuronal damage after IR, as neurons rely heavily on mitochondrial ATP production to support their high energy requirements (Pundik et al., 2012). Experimental evidence suggests that mitochondria are the main organelles that produce ROS within the cell, and under physiological conditions, they produce ATP through oxidative phosphorylation, maintain calcium homeostasis, and control antioxidant defense signals; while under pathological stress, they produce excess ROS and receive a large influx of calcium, opening the mitochondrial permeability transition pore (MPTP), which may lead to cell death (Bhatti et al., 2017; Wu et al., 2019).

Given that mitochondria act as energy centers and are important for cellular homeostasis, exploring the role of mitochondrial biogenesis as an endogenous protective response to ischemic injury may help us develop a strategy to enhance this beneficial effect and counteract the deleterious effects associated with ischemia. In previous studies, it was found that the PGC-1α signaling pathway is activated under transient global ischemia, which may trigger UCP2 and SOD2 expression and promote mitochondrial biogenesis in the CA1 region of the hippocampus, consistent with the role of mitochondrial biogenesis as a potential endogenous protective mechanism (Figure 1C; Yin et al., 2008; Vosler et al., 2009). Therefore, signaling pathways that promote upstream mitochondrial biogenesis, such as the PGC-1α signaling cascade response, may be new targets for therapeutic strategies targeting ischemic brain injury (Chen et al., 2010).

The ROS detoxification system and mitochondrial biogenesis may play an important role as endogenous protective mechanisms in cerebral ischemia. It has been demonstrated that increased mitochondrial biosynthesis can exert neuroprotective effects and reduce ischemic brain injury by increasing the number of mitochondria (Han et al., 2020). Previous studies have shown that PGC-1α is a powerful stimulator of mitochondrial biogenesis and gene transcription in cardiac, hepatic and skeletal muscle and neurological diseases and that PGC-1α activation or overexpression can be used to compensate for the neuronal mitochondrial loss in CIRI, where mitochondrial dysfunction and oxidative stress injury play a key role in the pathogenesis (Russell et al., 2004; Katsouri et al., 2012; Islam et al., 2018; Lee et al., 2019). The PGC-1α pathway enhances mitochondrial biosynthesis by eliminating ROS to enhance mitochondrial biogenesis and reduce brain injury. Two important ROS detoxifying proteins in mitochondria, UCP2 and SOD2, play a key role in neuronal fate and injury progression after ischemic stroke by regulating ROS production. PGC-1α is a major regulator of ROS scavenging enzymes, and PGC-1α reduces oxidative stress by activating its downstream UCP2 and SOD2 expression, thereby ameliorating neuronal injury (Yu et al., 2020). It has also been shown that upregulation of PGC-1α after stroke reduces the expression of pro-inflammatory cytokines such as microglia, suggesting that PGC-1α-induced mitochondrial biosynthesis not only maintains mitochondrial homeostasis but also attenuates mitochondrial dysfunction in CIRI through anti-oxidative stress and inflammatory responses (Han et al., 2021).

Neurons have high energy requirements and therefore mitochondrial homeostasis is critical for maintaining the normal neuronal function, and exploring the role of mitochondrial biosynthesis in response to ischemic injury may help to develop strategies to enhance this beneficial effect and ameliorate the pathological consequences associated with ischemia. Several studies have shown that ischemia disrupts mitochondrial structure and function, with significantly reduced mitochondrial respiratory function, increased levels of oxidative stress, and changes in mitochondrial permeability induced by calcium ion overload in Middle cerebral artery occlusion model (MACO) rats, ultimately leading to neuronal apoptosis in ischemic brain tissue (He et al., 2020). Previous studies have shown that enhancement of MB can reduce ischemic brain injury and improve neuronal damage by increasing the number and function of mitochondria (Valerio et al., 2011). MB requires coordinated expression of nuclear and mitochondrial genes, and PGC-1α is a co-transcriptional regulator that induces mitochondrial biosynthesis by activating the transcription factors TFAM and NRF1. Previous studies have shown that the PGC-1α signaling pathway is activated in transient ischemia and triggers mitochondrial biosynthesis in the CA1 region of the hippocampus, consistent with a protective effect through the enhancement of signaling pathways upstream of mitochondrial biosynthesis (Chen et al., 2010). It has been shown that activation of the PGC-1α pathway can protect ischemic neurons by promoting mitochondrial biosynthesis after CIRI to replace damaged mitochondria and exert mitochondrial protection (Lai et al., 2020).

Therefore, from the perspective of promoting PGC-1α-mediated mitochondrial biosynthesis and attenuating oxidative stress to protect mitochondrial function, it may be a promising therapeutic direction for neuroprotective treatment strategies against ischemic brain injury.

## 5. Conclusion

The ischemic–hypoxic state after cerebral ischemia leads to an imbalance in mitochondrial functional homeostasis. The massive generation of reactive oxygen species after reperfusion activates the expression of pro-oxidant enzymes, decreases the antioxidant capacity of neurons, and impairs mitochondrial function eventually leading to cell death. The massive inward flow of calcium ions in mitochondria and oxidative stress can lead to the opening of MPTP through lipid peroxidation and damage to the mitochondrial respiratory chain, resulting in a decrease in mitochondrial membrane potential and the release of substances such as pro-apoptotic proteins, which then activate apoptotic pathways and eventually lead to neuronal death (Jia et al., 2021). The organism can improve its dysfunction after cerebral ischemia by removing damaged mitochondria through autophagy and increasing new healthy mitochondria through MB. Therefore, the protection of mitochondrial function may be the focus of neuronal protection after CIRI.

Activation of the PGC-1α pathway and subsequent MB plays a protective role in CIRI, as PGC-1α reduces the release of ROS by increasing the expression of its downstream targets, such as UCP2 and SOD2, thereby improving neuronal damage and reducing apoptosis. It can also enhance the antioxidant and anti-inflammatory ability of neurons. Therefore, enhancing PGC-1α-mediated MB may be a new way to promote neurological recovery after cerebral infarction. Improving mitochondrial biosynthesis, promoting the replacement of damaged mitochondria in the cerebral ischemic zone, inhibiting oxidative stress and thus accelerating neurological recovery may be a new strategy for the treatment of CIRI and provide new ideas for the clinical treatment of IR.

## Author contributions

YY: conceptualization and writing—original draft preparation. YY, YT, HJ, LYC, and XMZ: writing—review and editing. JS, RP and YY: edited the final version of the manuscript, and JS and YY: participated in the drawing of the figures. All authors have read and agreed to the published version of the manuscript.

## Funding

This research was funded by the National Natural Science Foundation of China, grant number No. 81704188 and the Natural Science Research Program of the Hubei Provincial Education Department, grant number No. D20202006.

## Conflict of interest

The authors declare that the research was conducted in the absence of any commercial or financial relationships that could be construed as a potential conflict of interest.

## Publisher’s note

All claims expressed in this article are solely those of the authors and do not necessarily represent those of their affiliated organizations, or those of the publisher, the editors and the reviewers. Any product that may be evaluated in this article, or claim that may be made by its manufacturer, is not guaranteed or endorsed by the publisher.
